# Abstract of the Proceedings of the Buffalo Medical Association

**Published:** 1858-10

**Authors:** Austin Flint


ART. II.—*Abstract of Proceedings of the Buffalo Medical Association.*

TUESDAY EVENING, Sept. 7, 1858.

The Association met.

Present—The President, Dr. Wyckoff, in the chair; Drs. Gould, White, Newman, Rochester, Flint, Rogers, Stevens, Treat, Ring, Butler, Lemon, and Flint, Jr.

The minutes of the July and August meetings were read and accepted.

On motion, Drs. Rathbone and Coventry were invited to participate in the proceedings of the association until they could become members.

Prof. ROCHESTER presented the following interesting case of dislocation of the sternal extremity of the clavicle:

The patient was a laboring man 44 years of age, who was injured on the 28th of August, in the following manner. He was seated on a load of wood which he endeavored to drive under a bar; but miscalculating his distance, he was caught between the bar and the wood. The force which produced the dislocation was applied to the shoulder and was directed downwards and slightly backwards. This occurred about 5, P. M., and Prof. Rochester saw him shortly after. He then complained of pain, great difficulty of respiration and loss of speech. On examination it was found that the sternal extremity of the right clavicle was completely dislocated and thrown upwards and forwards, pressing against the pomum Adami. Reduction was immediately effected without difficulty, and a dressing was applied like that for a fracture of the clavicle. Prof. Hamilton saw the patient, and thought that there might be a dislocation of the lower extremity of the sternum from some of its cartilages; but it was impossible to determine exactly, whether this were the case, or the lower extremity of the sternum projected on account of the want of support by the clavicle at its superior extremity. The next day, Prof. Rochester was called to see the patient whom he found with a tumid abdomen, suppression of urine, and complaining of pain in the left sternal region. This at first led him to suspect more serious injuries, but he improved and is now doing well. Prof. Rochester employed a modification of the ordinary dressing for fractured clavicle, which was suggested to him by Prof. Moore, of Rochester. It consisted in a yoke of gutta percha accurately moulded, while warm, to the clavicle, and held in place by broad strips of adhesive plaster; he also employed a very broad and long strip of plaster extending around the body, to confine the arm in the desired position. This dressing answered the purpose exceedingly well. On measurement, the distance of each acromion process from the median line appears the same, and it is hoped that the cure will be as complete as we can hope for in such cases. A roller was applied over the adhesive straps, more with the object of protecting them, than in the hope that they would materially assist in keeping the parts in position.

Prof. ROCHESTER mentioned a pathological observation which he had made in making an autopsy. The patient was delivered twenty days before after a difficult and protracted labor. She had suffered from diarrhœa for two or three days before her confinement, and afterwards was attacked with dysentery, which terminated fatally on Sept. 6th. On post-mortem examination the uterus was found much larger than is usual at that time after delivery, and its cavity was occupied by a portion of adherent placenta, about three inches long by one inch broad and one in thickness. This was very closely adherent and it required considerable force to tear it away. There were no evidences of decomposition in the mass. The observation seemed interesting as showing the length of time a portion of placenta could be retained without undergoing disintegration. The portion of placenta which came away after delivery, was extruded spontaneously, and it seemed as though it had all been discharged.

Dr. Newman made an inquiry in respect to the dietetic treatment of dysentery. This disease had lately been prevalent in a certain section of the city, in an epidemic form. It had been the experience of Dr. Newman that ordinary broths and soups exerted a laxative effect. Beef essence even sometimes acted in the same way, but were better borne.

The subject was then discussed at some length by Drs. Wilcox, Wyckoff, Treat, Flint, Rochester and Newman. It was the general sentiment of of these gentlemen, that weak broths were apt to exert a laxative effect, but they all agreed in the propriety of sustaining patients reduced by dysenteric discharge, by beef essence, milk porridge, etc.; in different instances, of course, some of these forms of diet agreeing with the patient better than others.

Prof. Flint mentioned a case which had lately come under his observation, where there was no existing disease, but merely adhesions of the pericardium from an inflammation three years before. Last spring he was troubled with a little dyspepsia, and his attendant (an eclectic) reduced his diet to the lowest possible point. He was very much emaciated, having lost thirty pounds. The patient was 17 years old. Dr. Flint, finding no evidence of disease, recommended generous diet, much to the horror of the patient and friends, who, however, made the trial, with the most marked benefit. The patient has now been under this treatment for about a week, and is very much improved.

Dr. Treat mentioned a similar case. The patient was an old man who was thought to have heart disease, an apoplectic tendency, and a variety of disorders. He had been under homœopathic treatment, and was very much reduced by a rigid diet amounting to starvation. As soon as Dr. Treat had examined him, he was convinced that he was laboring under no disease, and ordered a venison steak; this treatment was followed up, and the patient recovered.

Prof. WHITE reported a case of inversion of the uterus of over fifteen years duration, which he had succeeded in returning. The patient was 32 years of age, and suffered the inversion when she was seventeen years old, after labor with her second child. Fourteen days after the accident, she was seen by Dr. White and inversio uteri was diagnosed. She has since been subject to repeated hæmorrhages and constant leucorrhœa, and is much reduced and anæmic. Reduction was effected on the 24th of Aug., in the presence of Drs. Flint, Rochester, Flint, Jr., and Mason. The time occupied in reduction was about fifty minutes. The patient was placed in the position before described, and chloroform given to moderate anæsthesia. The manipulations were the same as in the cases previously reported by Prof. White, and the difficulty of reduction was little, if any, greater than in the case of six months' standing. After the operation a speculum was introduced and all present saw the mouth of the uterus; the reduction was verified by measurements. She was then put to bed, and the fundus gently supported by a small rectum bougie for four hours. She improved rapidly, with no untoward symptoms during the succeeding eight days, and at the end of that time considered herself perfectly well. On the morning of the ninth day, however, after returning from breakfast, she imprudently strained considerably at stool, and was suddenly seized with violent pains in the abdomen. She was at the same time startled by the coming in of a friend while she was on the vessel. The mother and husband had left her a day or two before, considering her perfectly well. She immediately went to bed, seized with excruciating pain, and sent for Dr. White. She died on the seventh day, however, of peritonitis.

A post-mortem examination was made, six hours after death, in the presence of the gentlemen who were present at the reduction. A considerable quantity of liquid effusion, turbid and containing flocculi of lymph, was contained within the peritoneal cavity. The liquid was not measured; it would probably amount to from two to three pints. No fœcal odor, nor much fœtor on opening the abdomen. Collections of recently exudated lymph at different points between the convolutions of the intestines, and within the pelvic cavity. The intestines moderately tympanitic. Appendix vermiformis normal.

The uterus, with its appendages, having been removed, the exterior surface of the organ presented a few patches of soft, loosely adherent lymph; otherwise the appearance was normal. The organ was that of a normal uterus from the body of a person who has borne children. The os presented nothing abnormal. There was no trace of laceration anywhere. The structure of the organ and its inner surface, presented a healthy appearance. The ovaries were normal in size and exterior aspect.

Other organs not examined. No morbid appearances were observed exclusive of those which denoted recent, acute, general peritonitis.

Prof. Flint, was present at the reduction of the uterus by Prof. White. This was effected with great ease and under the moderate influence of chloroform. He regrets equally with Dr. White, the unfortunate accident which resulted in the death of the patient. The connection between the peritonitis and the operation did not seem to him to be very close; otherwise it would have supervened sooner. He did not think that it at all invalidates the success or propriety of the operation. The result will, perhaps, be thought to have more connection with the operation from an ordinary perusal of the case, than really exists. But a careful review of all the circumstances seems to show that the peritonitis was merely an unfortunate accident.

Prof. Rochester thought that there were some points which had not been sufficiently dwelt upon by Dr. White or Dr. Flint. The appearance of the uterus on examination after death, plainly indicated that no undue amount of force had been used in the operation. The exertion being exceedingly tedious and painful to the operator, Prof. White requested him to hold the uterus in place in the middle of the operation, to permit him to take a little rest. A very slight amount of force was necessary to accomplish this, and it seemed that a very little more, if applied for a short time longer, would be sufficient to reduce the organ. In regard to the occurrence of peritonitis, he also thought that it was a most unfortunate accident. Three-fourths of the cases which he had seen, occurred at this season of the year. There was no doubt but that the exhausting nature of the difficulty under which the patient had labored for so long, made the system more susceptible to the disease, and much less able to resist it. There was nothing in the appearance of the parts which would lead to the suspicion that the peritonitis commenced on the uterus; that it appeared to be a simple case of the disease. The greatest pain was referred to the epigastric region, and it was probable that this was its point of origin.

Prof. White mentioned a case of midwifery, in which he had applied the forceps on account of deformity of the pelvic bones. The patient was twenty years old, and in her third labor. In her previous confinements she had been delivered by the perforator. The pelvis was deformed by a narrowing of the pubic arch. Dr. Storck was in attendance and had applied the forceps, making all the traction he thought advisable. Dr. White thought that there was a possibility of delivery by carrying the head far back upon the perineum and os coxigis. He applied the instrument, and making traction downwards and backwards succeeded in delivering the woman of a large (about ten pounds) living male child. He had previously satisfied himself by auscultation, that the child was living. There was no laceration of the perineum, though it was put very much upon the stretch.

Prof. WHITE referred to the leading editorial article in the Buffalo Medical Journal, entitled "criminal abortions," making some remarks expressive of his concurrence in the views therein expressed, and offered the following resolutions, which he wished to lie on the table until the next meeting of the association.

*Resolved,* That the Association fully concurs in the sentiments expressed in the editorial article, entitled "Criminal Abortions," in the Sept. number of the Buffalo Medical Journal, relative to its frequency and criminality.

*Resolved,* That a committee of three be appointed from this Association to confer with the county and city authorities as to the laws now existing, if properly enforced, and whether further legislation is necessary for the abolishment of this great and growing evil.

*Resolved,* That this committee invite the coöperation of the Medical Societies and Associations in this State, in any measure which may be deemed necessary and expedient to lessen these horrible offences against the morality of the community.

On motion, these resolutions were laid on the table till the next meeting.

The Association then adjourned.

AUSTIN FLINT, JR., M. D.,
Secretary.

